# How to Improve the Curing Ability during the Vat Photopolymerization 3D Printing of Non-Oxide Ceramics: A Review

**DOI:** 10.3390/ma17112626

**Published:** 2024-05-29

**Authors:** Xiong Gao, Jingyi Chen, Xiaotong Chen, Wenqing Wang, Zengchan Li, Rujie He

**Affiliations:** Institute of Advanced Structure Technology, Beijing Institute of Technology, Beijing 100081, China; 3120226081@bit.edu.cn (X.G.); bitcjy0818@163.com (J.C.); xiaotongbit@163.com (X.C.); wangwq0628@163.com (W.W.); lzengchan@163.com (Z.L.)

**Keywords:** vat photopolymerization, non-oxide ceramics, curing ability, curing behavior

## Abstract

Vat photopolymerization (VP), as an additive manufacturing process, has experienced significant growth due to its high manufacturing precision and excellent surface quality. This method enables the fabrication of intricate shapes and structures while mitigating the machining challenges associated with non-oxide ceramics, which are known for their high hardness and brittleness. Consequently, the VP process of non-oxide ceramics has emerged as a focal point in additive manufacturing research areas. However, the absorption, refraction, and reflection of ultraviolet light by non-oxide ceramic particles can impede light penetration, leading to reduced curing thickness and posing challenges to the VP process. To enhance the efficiency and success rate of this process, researchers have explored various aspects, including the parameters of VP equipment, the composition of non-oxide VP slurries, and the surface modification of non-oxide particles. Silicon carbide and silicon nitride are examples of non-oxide ceramic particles that have been successfully employed in VP process. Nonetheless, there remains a lack of systematic induction regarding the curing mechanisms and key influencing factors of the VP process in non-oxide ceramics. This review firstly describes the curing mechanism of the non-oxide ceramic VP process, which contains the chain initiation, chain polymerization, and chain termination processes of the photosensitive resin. After that, the impact of key factors on the curing process, such as the wavelength and power of incident light, particle size, volume fraction of ceramic particles, refractive indices of photosensitive resin and ceramic particles, incident light intensity, critical light intensity, and the reactivity of photosensitive resins, are systematically discussed. Finally, this review discusses future prospects and challenges in the non-oxide ceramic VP process. Its objective is to offer valuable insights and references for further research into non-oxide ceramic VP processes.

## 1. Introduction

Compared to metal and polymer materials, ceramic materials possess several advantages, including a high hardness, high mechanical strength, low density, high melting point, excellent biocompatibility, and chemical stability. Consequently, ceramic materials hold significant promise for applications in aerospace [1,2,3], weaponry [4,5,6], biomedicine [7,8,9], and other fields. The rapid development of these sectors necessitates ceramic components with more intricate shapes and finer structures to achieve objectives like further weight reduction and enhanced load-bearing capacity [10,11]. However, the inherent hardness and brittleness of ceramics pose challenges for conventional machining techniques like drilling and milling. Furthermore, molding processes such as ceramic injection molding and gel casting suffer from drawbacks including low dimensional accuracy, high mold costs, and limited complexity when manufacturing components with intricate structures [12,13,14]. Fortunately, the emergence of additive manufacturing technologies based on the discretization–accumulation mechanism has provided new ways for fabricating ceramic components with complex shapes and fine structures. Numerous researchers have successfully fabricated ceramic components using techniques such as material extrusion (ME) [15,16,17], binder jetting (BJ) [18,19], selective laser sintering (SLS) [20,21,22], and vat photopolymerization (VP) [23,24,25]. Among these techniques, VP stands out for its high molding precision and excellent surface quality [24,25]. The VP process typically uses UV light as the source of curing energy. UV curing technology has the advantages of being highly efficient, environmentally friendly, energy-saving, enabling, and economical, as well as having a shorter curing time [26]. The above advantages make it widely applicable for oxide ceramics (e.g., Al_2_O_3_, ZrO_2_) [27,28,29,30] and bio ceramics (e.g., HA, TCP) [31,32]. The studies on slurry preparation [33,34], the optimization of VP process parameters [35,36], and debonding–sintering processes [37,38] for these ceramic materials have reached a mature stage and have been widely reported.

In comparison to oxide ceramics and bio ceramics, non-oxide ceramics typically consist of strongly covalently bonded compounds, resulting in higher melting points, superior mechanical properties, and enhanced chemical stability. Consequently, non-oxide ceramic materials find suitability in applications subjected to extreme conditions such as high temperatures, intense radiation, and high strain rates. Industries such as the petrochemical, mechanical manufacturing, nuclear, and semiconductor industry have extensively employed non-oxide ceramic materials, specifically carbides (e.g., SiC, B_4_C) [39,40,41], nitrides (e.g., Si_3_N_4_, BN) [42,43,44], and borides (e.g., ZrB_2_) [45,46,47]. However, non-oxide ceramics, characterized by their darker coloration, exhibit a higher refractive index and absorbance. Consequently, non-oxide ceramics demonstrate enhanced light extinction properties. In the VP process, this increased light extinction capability results in a reduced light penetration depth within the non-oxide ceramic slurries, leading to a diminished cured thickness. Typically, oxide ceramic slurries and bio ceramic slurries readily achieve curing thicknesses exceeding 200 μm due to their weaker extinction characteristics [34,48]. Conversely, unmodified non-oxide ceramic slurries typically yield curing thicknesses of less than 50 μm [49,50]. Such lower curing thicknesses present numerous challenges for the non-oxide ceramic VP process. On the one hand, a lower curing thickness results in weak interlayer bonding, rendering it highly susceptible to the formation of defects like cracks and pores during molding, debonding, and sintering processes [51]. On the other hand, a lower curing thickness prolongs the molding time, leading to a substantial reduction in manufacturing efficiency. Moreover, as a suspension, the stability of the ceramic photosensitive resin slurry is significantly challenged during excessively long molding processes [52,53]. The use of ceramic precursors as raw materials for the VP process can circumvent these problems to some extent. Instead of non-oxide ceramic particles, such VP slurries contain ceramic precursors such as polysiloxane [54] and polycarbosilane [55]. Subsequently, at high temperatures, the precursor undergoes pyrolysis, resulting in polymer chain breakage, cross-linking, and ultimately the formation of polymer-derived ceramics (PDCs). However, this process has limitations related to ceramic conversion. This often leads to PDCs with lower densities and weaker strengths, rendering them unsuitable for applications that require high strength or other mechanical properties. Furthermore, when ceramic precursors are used for pyrolysis to produce non-oxide ceramics like SiC and Si_3_N_4_, certain impurity ceramic phases such as SiOC and SiCN may be present in the final products, which can affect their performance. 

From an economic standpoint, precursors with high ceramic conversion rates tend to be expensive, thus limiting the widespread adoption of this process on a larger scale. Therefore, the utilization of non-oxide ceramic particles to prepare slurries followed by VP processing for part fabrication remains an important area of research. In view of this, several researchers have already investigated the light-curing behavior of non-oxide ceramic slurries. Ding et al. [49] improved the cross-linking ability of the polymer by increasing the reactivity of the photosensitive resin, which in turn improved the curing behavior of a SiC ceramic slurry. Liu et al. [56] observed that when the size of Si_3_N_4_ particles increased, the absorbance of the particles decreased, which was found to be beneficial for enhancing the curing ability of the slurry. In addition, Tang et al. [57] introduced low-absorbance SiO_2_ particles into a SiC slurry, resulting in an augmented cured thickness with increasing SiO_2_ particle percentages. It is evident that the curing process has emerged as a central challenge in the non-oxide ceramic VP process, garnering significant attention.

Although all of the aforementioned studies have improved the curing behavior of the non-oxide ceramic VP process to some extent, several limitations and unresolved scientific issues persist. The VP curing process of ceramic slurries is influenced by various factors, encompassing the parameters of the VP equipment, the characteristics of ceramic particles, and the reactivity of the photosensitive resin. Particularly in the case of non-oxide ceramic slurries, alterations in the absorbance and refractive index of ceramic particles can induce significant changes in the VP curing process. Thus, attaining a comprehensive understanding of the light-curing behavior of non-oxide ceramic slurries is imperative. This review initially delineates the curing mechanism of the ceramic VP process, elucidating the chain initiation, chain polymerization, and chain termination processes of photosensitive resins. Furthermore, the diverse interactions between light and ceramic particles in non-oxide ceramic VP slurries have been introduced, encompassing absorption, scattering, and reflection phenomena. Subsequently, pivotal factors influencing the light-curing behavior of non-oxide ceramic pastes are systematically explored. These factors include the wavelength and power of incident light, particle size, volume fraction of ceramic particles, refractive indices of photosensitive resins and ceramic particles, incident light intensity, critical light intensity, and reactivity of photosensitive resins. The approaches employed by researchers to optimize the light-curing behavior of non-oxide ceramic pastes are subsequently summarized, utilizing the aforementioned influencing factors as a framework for classification. Finally, challenges and prospects regarding the further development of the non-oxide ceramic VP process are deliberated upon. The authors aspire that this review will furnish valuable insights and references for the advancement of research pertaining to the VP process in non-oxide ceramics.

## 2. Curing Mechanism during the Vat Photopolymerization 3D Printing of Ceramics

**Figure 1:**
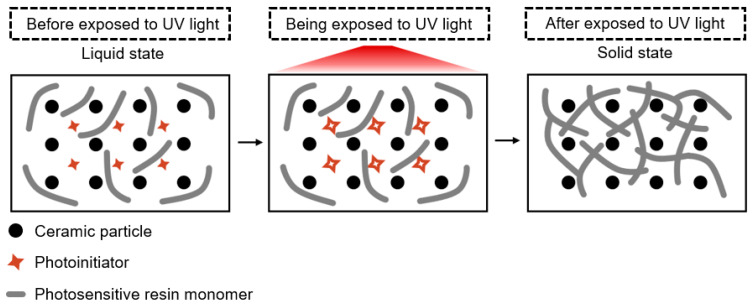
Schematic of the composition and UV light-curing process of a ceramic VP slurry.

Before discussing any issues related to the ceramic VP process, it is important to first understand the raw material composition and curing mechanism inherent to it. Ceramic VP slurries typically comprise ceramic particles, photosensitive resin monomers (and/or oligomers) with functional groups, photoinitiators, dispersants, and other additives [58]. Figure 1 depicts a comprehensive overview of the composition of the ceramic VP slurry and the UV light-curing process. The ceramic particles remain in a relatively stable state within the photosensitive resin in the presence of dispersants, which makes the whole system behave as a suspension with a certain viscosity [59,60]. The photoinitiator plays a crucial role in enabling light-induced curing within this suspension system. When exposed to UV light, the photoinitiator undergoes activation, initiating cross-linking polymerization of the photosensitive resin [61]. Concurrently, the ceramic particles become encapsulated within the polymer matrix, resulting in the formation of ceramic green parts. This process primarily involves a chain polymerization reaction of the photosensitive resin monomers. To facilitate comprehension of this process, it is delineated below into three key steps: chain initiation, chain polymerization, and chain termination.

When ceramic VP slurries are exposed to UV light, the photoinitiator absorbs light energy, transitioning from the ground state (PI) to the excited state (PI*), thereby generating active radicals or cations [62]. Photoinitiators that are reliant on cationic polymerization demonstrate excessive initiation rates [49], rendering them unsuitable for achieving higher-resolution ceramic green parts. Consequently, the photoinitiators utilized in the ceramic VP process typically operate via radical polymerization. These active radicals (R*) possess the ability to attack the C-C double bonds within the photosensitive resin monomer (M), bestowing upon the monomer chains the requisite reactivity for cross-linking, thereby culminating in the completion of the chain initiation stage [63]. Equation (1) shows the transition of the photoinitiator from the ground state to the excited state. Equation (2) shows the generation of active radicals by the photoinitiator in the excited state. Together, these two equations demonstrate the chain initiation stage of the reaction.

(1) $$\mathrm{PI}\overset{\mathrm{hv}}{⟶}{\mathrm{PI}}^{*}$$

(2) $${\mathrm{PI}}^{*}⟶R_{1}^{*}{+R}_{2}^{*}$$

Subsequently, the chain polymerization stage is initiated. The reaction equation for this stage is depicted in Equation (3). Successive interactions between active chain termini and monomeric units lead to the elongation of polymer chains. This elongation process occurs concomitantly with the polymerization of the excited carbon–carbon double bonds [63]. As these reactive chains continue to elongate until all are cross-linked and the radicals lose their activity (R), the chain polymerization stage reaches its conclusion.

(3) $$R^{*}+M⟶{R(M)}_{n}^{*}$$

Equation (4) shows the entire chain polymerization reaction to the chain termination stage, wherein polymer chains assemble into a highly crosslinked network structure. This process, when viewed on a macroscopic scale, signifies the completion of light curing, resulting in ceramic green parts with a certain mechanical strength.

(4) $${R(M)}_{n}^{*}{+R(M)}_{m}^{*}⟶{R(M)}_{m+n}R$$

Based on the above description of the light-curing mechanism during the ceramic VP process, it can be concluded that only the photoinitiators in the slurry that receives UV light energy will be excited, which in turn causes the curing of the slurry system. However, photoinitiators that are not exposed to UV light are not excited. Therefore, during the ceramic VP process, the penetration depth of UV light in the slurry plays a crucial role in determining the slurry’s light-curing behavior. The various interactions between UV light and ceramic particles in a non-oxide ceramic VP slurry influences the depth of light penetration to different degrees. This will be discussed in detail in Section 3.

## 3. Light–Particle Interactions during the Vat Photopolymerization 3D Printing of Ceramics

When UV light is incident into the ceramic VP slurry, its energy gradually decreases with increases in the penetration depth. This is due to the fact that various interactions between light and ceramic particles weaken the intensity of transmitted light, which in turn impede the propagation of UV light. Once the light energy decays to zero, the penetration depth of the light will no longer continue to increase. Beer’s law [64] appropriately describes the attenuation of light energy with respect to penetration depth:

(5) $$E=E_{0}•\exp(-\gamma L)$$

where E is the energy density, $E_{0}$ is the initial energy density, γ is the extinction coefficient, and L is the penetration depth of the UV light. To provide a more precise depiction of UV light attenuation across different ceramic VP slurries and its consequential impact on curing behavior, elucidation of the diverse light–particle interactions during the VP process is imperative. 

**Figure 2:**
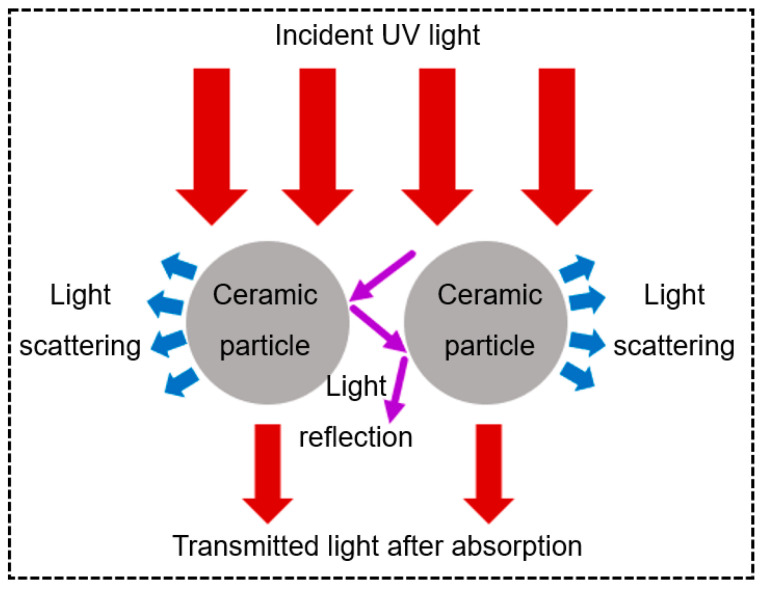
Light–particle interactions during the ceramic VP process.

As shown in Figure 2, these interactions encompass light absorption, light scattering, light reflection, and light transmission, with reflection representing a distinct manifestation of scattering. The absorption, scattering, and reflection of light by ceramic particles collectively influence the transmission and penetration depth of UV light, which is reflected as γ and L in Equation (5). Section 3.1 discusses the light absorption by ceramic particles. Increased light absorption by ceramic particles correlates with diminished UV light penetration depths. Concurrently, the competition between light absorption by ceramic particles and the photoinitiator diminishes the energy available for photoinitiator excitation, potentially resulting in a reduced curing thickness of the ceramic VP slurry. Section 3.2 addresses the light scattering by ceramic particles. Pronounced scattering not only engenders light energy loss but also induces blurring at the cured area periphery, thereby compromising the resolution of the ceramic VP process. Subsequently, Section 3.3 elucidates the light reflection by ceramic particles. An elevated intensity of reflected light on ceramic particle surfaces, coupled with low impediments to further light propagation, augments the UV light penetration depth within the ceramic VP slurry, consequently enhancing the curing thickness.

### 3.1. Light Absorption by Ceramic Particles

In non-oxide ceramic slurries, it is common for photosensitive resins to possess colorless and transparent properties, while dispersants are typically added in small quantities (generally less than 10 wt.% relative to ceramic particles). The absorption of light by these components cannot significantly impact the light-curing capability of slurry. However, the photoinitiators contribute to the light-curing ability, even though they are also present in relatively small amounts. However, the presence of ceramic particles leads to significant light energy absorption, which reduces the penetration depth of light in the slurry and at the same time creates a competing relationship with the absorption of light by the photoinitiators. The light absorption ability of the non-oxide ceramic particles is significantly stronger than that of the oxide ceramic particles, which leads to a smaller penetration depth of light in the non-oxide ceramic VP slurry, which in turn deteriorates the curing behavior of the slurry. To gain clarity on the influence of light absorption in the light-curing behavior of the non-oxide ceramic VP slurry, it becomes necessary to understand the mechanisms of light absorption by ceramic particles and subsequently implement targeted improvements.

Light is a special form of electromagnetic waves. The absorption mechanisms of materials for electromagnetic waves of different frequencies vary, but these mechanisms are mostly related to various forms of energy level transitions. The light sources used in the ceramic VP process are based on UV or violet light. The absorption of materials for ultraviolet–visible light is related to the energy level transition of outer electrons between the valence band and the conduction band [65,66]. When a substance exhibits a strong absorption capability for ultraviolet–visible light, it indicates that the energy required for the outer electrons to transition from the valence band to the conduction band is smaller, i.e., the band gap is narrower, resulting in stronger absorption of light. The band gap is primarily determined by the material’s band structure and is influenced by factors such as the crystal structure and atomic bonding properties. The cause of the band gap involves knowledge related to physics, which is not the focus of the discussion here. Therefore, this review only discusses the effects of band gap on the ability of ceramic particles to absorb ultraviolet–visible light. For example, the impact of band gap on the light absorption capability of SiC, Si_3_N_4_, and SiO_2_ are discussed. Table 1 illustrates their respective band gaps, colors, and the curing thickness of the VP slurries. Among these materials, SiO_2_ has the widest band gap and hence the weakest light absorption capability. This manifests as the lightest color, the lowest ultraviolet absorption, and a curing thickness of up to several hundred micrometers [48]. In contrast, the band gaps of SiC and Si_3_N_4_ are narrower, making their outer electrons more prone to absorbing the energy of ultraviolet–visible light, thereby undergoing energy level transitions. In this scenario, the curing thickness of SiC and Si_3_N_4_ ceramic VP slurries can be as low as tens of micrometers [49,50]. It can be concluded that ceramic particles with narrower band gaps exhibit stronger light absorption capabilities, leading to strict limitations on the penetration depth of light. Additionally, the photoinitiator can only receive a very limited amount of light energy. Consequently, the curing thickness of ceramic VP slurries will significantly decrease.

### 3.2. Light Scattering by Ceramic Particles

A non-oxide ceramic VP slurry is essentially a suspension. The refractive index difference between the ceramic particles and the photosensitive resin results in a certain degree of light scattering when the slurry is exposed to light [67,68]. This scattering phenomenon leads to light loss and reduces the penetration depth, consequently impacting the curing thickness. Furthermore, significant scattering can result in blurring or distortion at the edges of the cured region, thereby compromising the dimensional accuracy of the VP process [69].

The scattering behavior of light in suspensions has been discussed in various theories. In each of these theories, a dimensionless function Q, called the extinction coefficient efficiency, is introduced to characterize the degree of light loss by scattering [70]. Q is related to the solid loading ϕ, particle size d, and the ratio $m=n_{p}/n_{0}$:

(6) $$Q(\phi ,x,m)={\left(m-1\right)}^{2}\tilde{Q}(\phi ,x,m)={\left(\Delta n/n_{0}\right)}^{2}\tilde{Q}(\phi ,x,m)$$

where x=πd/λ, $n_{p}$ is the particle refractive index, $n_{0}$ is the solution (specifically referring to photosensitive resins in the VP process) refractive index, and Δn is the difference between the two.

Q has been explored in more depth according to different scattering theories. There are several equations for Q in terms of x and ρ, where ρ is related to the refractive index difference between the ceramic and the UV-curable solution [70].

(7) $$\rho =x\Delta n=x(n_{p}-n_{0})$$

When the particle size is smaller than the wavelength of the incident light, Rayleigh scattering theory [70,71] suggests the following:

(8) $$Q_{ray}=\frac{1}{2}\rho^{2}=\frac{1}{2}x^{2}\Delta n^{2}$$

In addition, the Mie scattering theory [70,72] is not limited by particle size and the wavelength of incident light. Therefore, Q has also been discussed using the Mie scattering theory. For Mie scattering, the following applies:

(9) $$Q_{mie}=2-\frac{4\sin\rho }{\rho }+\frac{4}{\rho^{2}}(1-\cos\rho )$$

Based on these theories, it becomes evident that the disparity in the refractive index between ceramic particles and photosensitive resins significantly impacts light scattering, ultimately resulting in losses of incident light energy to varying degrees. Figure 2 also shows the light scattering by different ceramic particles. It can be seen that the larger the Δn value, the stronger the scattering effect, and the more unfavorable it is for light to propagate in the direction of incidence.

Generally, photosensitive resins possess a refractive index of around 1.4–1.5 [33,73,74,75]. However, ceramic particles commonly exhibit refractive indices exceeding this range. For instance, Table 2 shows the refractive index of typical oxide and non-oxide ceramic particles used in the VP process. It is observed that based on the discussion of Q, an increasing difference in the refractive index between ceramic particles and photosensitive resins leads to a decrease in scattered light intensity. Consequently, the overall intensity and depth of light penetration are reduced, thereby compromising the curing capability of the slurry.

### 3.3. Light Reflection by Ceramic Particles

Light reflection is a special form of light scattering. Despite the relatively low reflectivity of ceramic materials compared to metal materials, the light reflection phenomenon when light interacts with a ceramic VP slurry still cannot be ignored. When the surface of the ceramic particles reflects light and the reflected light is able to continue to propagate, it contributes to an increase in the depth of light penetration.

In this regard, non-oxide ceramic slurries exhibit significant differences compared to oxide ceramic slurries. Firstly, as discussed in Section 3.1, non-oxide ceramic particles have a higher light absorption capacity than oxide ceramic particles, leading to reduced reflection at the ceramic particle–photosensitive resin interface. Additionally, oxide ceramic particles can typically be prepared as spherical or nearly spherical, while non-oxide ceramic particles tend to have irregular shapes and fragmented structures and are difficult to prepare into spherical shapes via secondary processing. As shown in Figure 2, reflected light can propagate relatively easily to greater depths between nearly spherical particles. However, for non-oxide ceramic particles, the rough surfaces and irregular shapes impede the escape of reflected light [67], thereby limiting the further propagation of light within the VP slurry. As a result, for non-oxide ceramic VP pastes, the overall reduction in reflected light is accompanied by a rise in the difficulty of reflected light escape, which results in a notable restriction in the depth of UV light penetration. Consequently, this limitation ultimately leads to a reduction in the cured thickness.

## 4. Key Factors Affecting the Light-Curing Behavior of Non-Oxide Ceramic VP Slurries

To investigate the light-curing behavior of non-oxide ceramic VP slurries, it is crucial to establish a qualitative or quantitative correlation between the curing behavior and the factors present in the slurry components. The Jacobs equation [78] is widely employed as a method for determining the curing thickness of VP slurries:

(10) $$D_{c}=D_{p}•\ln\left(\frac{E_{0}}{E_{crit}}\right)$$

where $D_{c}$ is the curing thickness, $D_{p}$ is the depth of light penetration in the ceramic VP slurry, $E_{0}$ is the incident light intensity, and $E_{crit}$ is the critical light intensity. In addition, Griffith et al. [70] proposed a correlation between the depth of penetration and the factors present in the slurry components:

(11) $$D_{p}\propto \frac{2d}{3}\frac{\lambda }{\phi }\frac{n_{0}^{2}}{\Delta n^{2}}$$

where d is the average particle size of the ceramic particles, λ is the wavelength of incident light, ϕ is the volume fraction (i.e., solid loading) occupied by the ceramic particles, $n_{0}$ is the refractive index of the photosensitive resin, and Δn is the difference between the refractive indices of the ceramic particles and the photosensitive resin. Associating Equation (10) with Equation (11) yields Equation (12).

(12) $$D_{c}\propto \frac{2d}{3}\frac{\lambda }{\phi }\frac{n_{0}^{2}}{\Delta n^{2}}\ln\left(\frac{E_{0}}{E_{crit}}\right)$$

Changes in the several physical quantities on the right side of the equation result in varying degrees of impact on the curing thickness. Therefore, these physical quantities are defined as the several key factors that affect the light-curing behavior of non-oxide ceramic VP slurries. To assess the magnitude of influence exerted by these factors, they are categorized and discussed based on primary terms, quadratic terms, and terms related to the logarithmic calculation. In the first place, d, λ, and ϕ are primary terms, meaning that a change in any of these factors leads to a moderate alteration in the curing thickness. Next, both $n_{0}$ and Δn are quadratic terms, meaning that a change in either of these factors leads to a relatively large change in the curing thickness. Finally, both $E_{0}$ and $E_{crit}$ are terms related to the logarithmic calculation. Only when $E_{0}/E_{crit}>1$ is the logarithmic value positive at this time the system starts curing. Therefore, the effects of changes in these factors on the curing thickness are initially moderate and then rapidly diminish. 

In addition, the reactivity of the photosensitive resin [79] is another important factor that influences the light-curing behavior of non-oxide ceramic VP slurries, although it is not mentioned in Equation (12). It is determined by the functionality of the photosensitive resin, the concentration of the photoinitiators, and the initiating ability of the photoinitiators. Therefore, the following discussion includes an analysis of the reactivity of the photosensitive resin.

### 4.1. Ceramic Particle Size

Commercially available non-oxide ceramic particles exhibit a wide range of particle sizes, ranging from the nanometer scale to the micrometer scale. This variability allows for the adjustment of particle size in ceramic VP slurries. However, not all particle sizes are suitable for use as raw materials in VP slurries. According to Equation (12), an increase in ceramic particle size results in an increase in the curing thickness. Therefore, excessively fine powders are generally avoided as raw materials for VP slurries. On the other hand, using excessively coarse powders is also not recommended, as inappropriate particle sizes can negatively impact the slurry’s stability, rheological properties, and the sintering performance of the final product [80].

**Figure 3:**
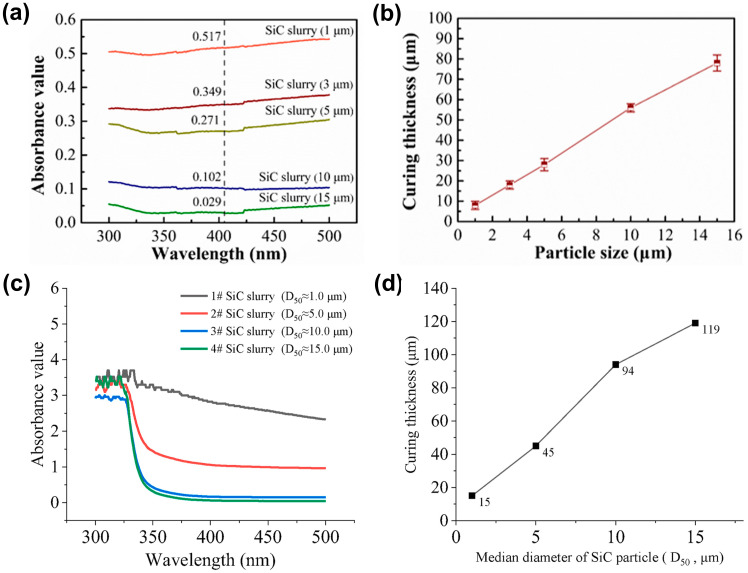
(**a**) Absorbance values and (**b**) curing thickness curves of SiC slurries with different particle sizes. Adapted from ref. [49]. (**c**) Absorbance values and (**d**) curing thickness curves of SiC slurries with different particle sizes. Adapted from ref. [81].

Some researchers have optimized the light-curing behavior of non-oxide ceramic slurries based on the approach of adjusting the ceramic particle size. Specifically, Ding et al. [49] prepared VP slurries with a solid loading of 30 vol% by utilizing SiC particles of different sizes (1, 3, 5, 10, and 15 µm). The absorbance values and curing thicknesses of the SiC slurries with different particle sizes are shown in Figure 3a,b. It was observed that the absorbance values significantly increased as the particle size decreased, while the curing thickness decreased accordingly. At a particle size of 15 µm, the slurry achieved a curing thickness of 78 μm. However, when using fine particles with a size of only 1 µm, the resulting curing thickness was only approximately 8 μm. Similarly, Hu et al. [81] formulated VP slurries with a solid loading of 40 vol% using SiC particles with D50 values of 1, 5, 10, and 15 µm and obtained analogous conclusions. Figure 3c,d illustrate the absorbance values and curing thickness of SiC slurries with different particle sizes. With an increase in particle size, the curing thickness of the slurry also increased, ranging from 15 µm to 119 µm. These results indicate that larger particle sizes diminish the extinction capacity of the solid phase.

Although an increase in particle size can lead to an increase in curing thickness, it can also lead to a decrease in the stability of the slurry and a reduction in the sintering performance of the green part. The destabilization of the slurry occurs due to the settling of ceramic particles in suspension. The free settling velocity of ceramic particles can be determined by applying Stokes’ law [82] to analyze their settling under the influence of gravity. Therefore, the following equation is derived:

(13) $$u_{0}=\frac{(\rho_{p}-\rho )g}{18\mu }d^{2}$$

where $u_{0}$ is the free settling velocity of the particles, d is the particle size, $\rho_{p}$ is the density of the particles, ρ is the density of the photosensitive resin, g is the gravitational acceleration, and μ is the viscosity of the slurry. The deterioration of slurry stability is evident as the particle size increases. Additionally, coarse particles exhibit lower surface energies, resulting in lower sintering activities [83]. The green parts prepared via the VP process contain a substantial amount of organic matter, which significantly reduces the density after debinding and results in the presence of more pores. Inadequate sintering activities of the ceramic particles can lead to a low relative density of the final product and diminished mechanical properties. Consequently, using overly coarse particles as raw materials for VP slurries is not advisable.

However, fine particles not only result in enhanced light extinction but also possess a higher specific surface area and surface energy. This leads to a stronger van der Waals force effect between the fine particles, increasing their tendency to agglomerate and causing the viscosity of the light-curing slurry to rise [84]. In the VP process, the movement of the photosensitive resin monomers and the free radicals or cations generated by the excited photoinitiators determine the initiation and termination of the light-curing reaction. An increase in slurry viscosity hinders the movement of these species, directly impacting the diffusion ability of the substances involved in the light-curing reaction and adversely affecting the light-curing behavior. Moreover, excessively high slurry viscosities can impede the uniform spread of each new layer during the VP process, introducing numerous defects into the green parts and compromising the quality of the final product [85]. 

**Figure 4:**
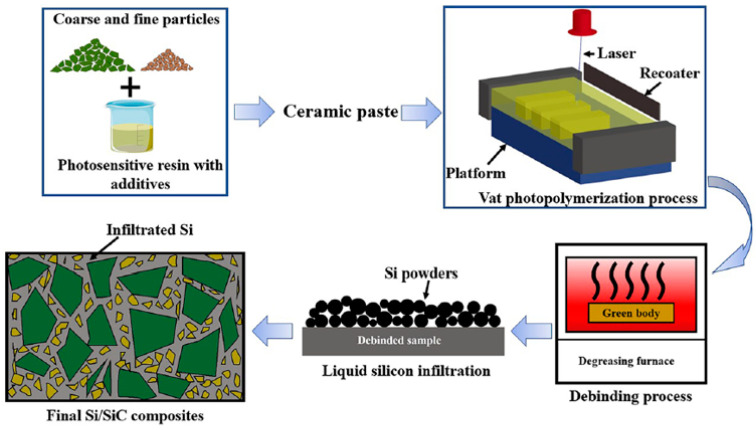
Schematic diagram of the preparation process of Si/SiC composites. Adapted from ref. [86].

As mentioned above, it is not recommended to use either coarse or fine particles alone as raw materials for non-oxide ceramic VP slurries. Several researchers have investigated the effects of particle gradation, i.e., the utilization of a combination of coarse and fine particles, in a certain ratio. For example, Tang et al. [86] employed two SiC particle sizes with D50 values of 58,626 µm and 3289 µm for grading. In addition, they incorporated carbomer as a thickener. The addition of a thickener is imperative to ensure the suspension stability of the larger particles within the slurry and prevent their settling, despite being rarely utilized in other non-oxide ceramic VP slurry formulations. This SiC VP slurry has a high solid loading of 60 vol%. However, the curing thickness can reach up to 130 μm. The utilization of particle grading enables the inclusion of larger particle sizes, thereby further diminishing the extinction capacity of the solid phase while maintaining a high solid loading. However, the oversized particles significantly impeded sintering activity. Therefore, the LSI process was employed to ensure the dense sintering of the products. Eventually, Si/SiC composites were prepared. Figure 4 is the schematic diagram of the preparation process of Si/SiC composites utilizing a particle-graded slurry and LSI process.

### 4.2. Wavelength of Incident Light

According to Equation (12), increasing the wavelength of the incident light results in an increase in the curing thickness. Conventional UV curing processes use mercury lamps, electrode-free lamps, excimer lamps, or LEDs as light sources [26]. These light sources emit UV light of different wavelengths. According to Planck’s formula, shorter wavelengths of light have higher energies:

(14) $$E=hv=h\frac{c}{\lambda }$$

where E is the energy of light, h is Planck’s constant, ν is the frequency of light, c is the speed of light, and λ is the wavelength of light. When the wavelength of light exceeds the range of UV or violet light, it leads to a reduction in the energy of light, which can hinder light penetration and the light-curing reaction. Additionally, higher-wavelength light is difficult to focus, resulting in a significant decrease in the accuracy of the VP process. For reasons of printing accuracy, safety, and longevity, the light sources used in the VP process are usually 405 nm digital light projectors (for DLP applications) [30,35] or 355 nm lasers (for SLA applications) [59,79]. Furthermore, the wavelength of the light source is typically fixed for a VP device, and photoinitiators are specifically designed to absorb particular wavelengths of UV or violet light [87,88,89]. Consequently, adjusting the wavelength of the incident light to control the curing thickness of the slurry is costly and complex, and the effect is generally not significant. Therefore, this method is not common.

### 4.3. Volume Fraction of Ceramic Particles

Equation (12) states that reducing the volume fraction of ceramic particles leads to an increase in the curing thickness. There are a few reasons for this effect. Firstly, when the volume fraction of ceramic particles decreases, more light can be absorbed by the photoinitiators. This results in the generation of more reactive radicals or cations, which promotes the curing reaction and increases the curing thickness. Secondly, with a lower volume fraction of ceramic particles, there is less “blockage” of light as it travels through the suspension. The absorption, scattering, and refraction of light by the ceramic particles become weaker, which allows for increased penetration of light into the slurry and further contributes to the increase in curing thickness.

**Figure 5:**
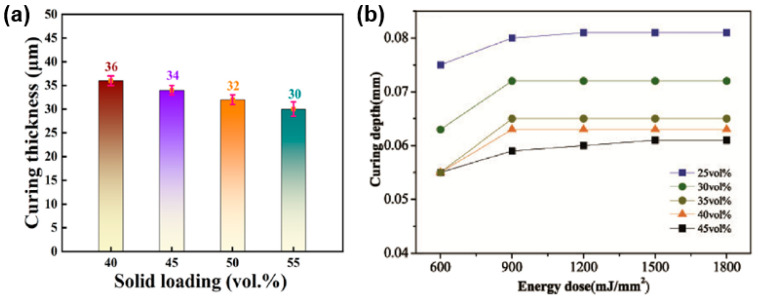
(**a**) Curing thickness of Si_3_N_4_ slurries with different solid loadings. Adapted from ref. [50]. (**b**) Dependence of the curing depth on the exposure energy for ceramic slurries with different Si_3_N_4_ solid loadings. Adapted from ref. [56].

Numerous studies focusing on the VP process of non-oxide ceramics have consistently corroborated the assertion that reducing the solid loading of the slurry leads to an increase in the curing thickness. For instance, Dong et al. [50] prepared Si_3_N_4_ slurries with solid loading of 40, 45, 50, and 55 vol%. These slurries were exposed to a light intensity of 10,000 μW/cm^2^ for a duration of 6 s. As the solid loading increased, the curing thickness of the slurry decreased from 36 µm to 30 µm. Similarly, Liu et al. [56] developed Si_3_N_4_ slurries with solid loading varying from 25 vol% to 45 vol% and analyzed the resulting curing thickness. They concluded that there was an inverse relationship between the solid loading and the curing thickness, as shown in Figure 5a,b.

While reducing the volume fraction of ceramic particles may increase the curing thickness, it can create additional challenges during the debinding and sintering process. A lower solid loading makes the debonding process more difficult, as it may result in the creation of more defects [51]. Additionally, it can lead to a lower relative density, requiring more complex subsequent densification processes to achieve the desired product density. These additional steps can increase the complexity and cost of the overall process.

### 4.4. Refractive Index of Photosensitive Resins and Ceramic Particles

When the quadratic terms in Equation (12) are changed, the curing thickness experiences a more pronounced variation. Therefore, the refractive index becomes a crucial factor influencing the curing thickness of the ceramic VP slurry. Table 3 presents the relevant parameters of commonly used photosensitive resins in ceramic UV slurries. It can be observed that the tuning range for the refractive index of photosensitive resins ($n_{0}$) is limited, which usually ranges from 1.4 to 1.5. Consequently, the focus should be on reducing the refractive index of ceramic particles to achieve a lower Δn value.

As depicted in Table 2, the refractive index of oxide ceramic particles is lower than that of non-oxide ceramic particles. Consequently, certain researchers have substituted a portion of the non-oxide ceramic particles in the slurry with oxide ceramic particles. This intervention has yielded a reduced refractive index of the ceramic particles in the VP slurry, thereby leading to a lower Δn value. As a result, this optimization has profoundly improved the light-curing behavior and increased the curing thickness.

**Figure 6:**
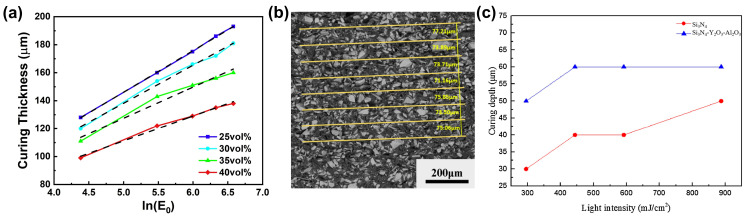
(**a**) Curing thickness of the SiC slurries with different SiC ratios; (**b**) SEM micrograph for cross-section of the green part. Adapted from ref. [57]. (**c**) Curing depth of slurries with Si_3_N_4_ and Si_3_N_4_-Y_2_O_3_-Al_2_O_3_. Adapted from ref. [99].

For instance, Tang et al. [57] employed various ratios of SiC and SiO_2_ particles to develop VP slurries with a fixed solid loading. The incorporation of a substantial amount of SiO_2_ particles, which possess a low extinction capacity, significantly reduced the refractive index difference between the ceramic particles and the photosensitive resin. As a result, reducing the percentage of SiC particles led to a notable increase in the curing thickness of the slurry. As shown in Figure 6a, a curing thickness of 100–200 μm was achieved, ensuring a reliable curing accuracy. Finally, they set the printing thickness to 75 μm and then prepared SiC ceramic green parts via the VP process. From Figure 6b, it can be seen that the average thickness of the actual printing layer is 74.34 μm, which is consistent with the design value. On the other hand, Chen et al. [99] formulated a Si_3_N_4_ VP slurry with a solid loading of 50 vol% and introduced 10 wt.% of Y_2_O_3_-Al_2_O_3_ particles. Figure 6b revealed that the pure Si_3_N_4_ slurry had a curing thickness of 30–50 μm, whereas the addition of Y_2_O_3_-Al_2_O_3_ particles resulted in a curing thickness of 50–60 μm.

**Figure 7:**
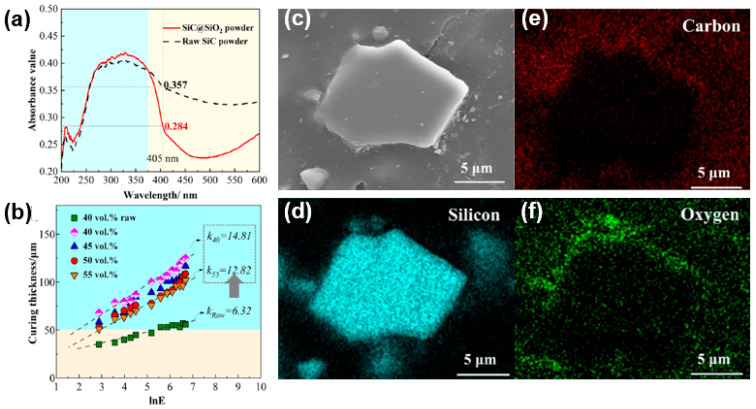
(**a**) Absorbance value of raw SiC and pre-oxidized SiC@SiO_2_ ceramic particles; (**b**) curing thickness of SiC and SiC@SiO_2_ ceramic slurries; (**c**) SEM micrograph of a SiC particle after oxidation; (**d**–**f**) EDS analysis of the SiC ceramic particle in (**c**). Adapted from ref. [100].

While the introduction of a significant number of oxide ceramic particles has been shown to improve curing behavior, this approach results in a residual oxide ceramic phase that can be difficult to remove using subsequent processes [86]. To reduce the oxide ceramic phase residue while maintaining a desired curing thickness, researchers have explored the application of surface coatings on non-oxidized particles. For instance, Cao et al. [100] subjected 10 µm SiC particles to oxidation at 1200 °C for 4 h, resulting in the formation of SiC@SiO_2_ core–shell-structured particles with an unchanged particle size. Figure 7a illustrates the absorbance value of raw SiC and pre-oxidized SiC@SiO_2_ ceramic particles. The SiO_2_ shell layer effectively reduced the particle absorbance from 0.357 to 0.284. As shown in Figure 7b, the slurry formulated with untreated SiC particles exhibited a curing thickness of only around 50 μm at a solids content of 40 vol%. In contrast, the slurry formulated with SiC@SiO_2_ particles achieved a curing thickness of up to 120 μm. Even if the solid loading is increased to 55 vol%, the curing thickness can still be as high as about 100 μm. Figure 7c–f illustrate the SEM micrograph and EDS analysis of SiC particles after oxidation. They show that a SiO_2_ layer with a thickness of less than 1 μm was formed on the surface of SiC particle, as evidenced by the distribution of oxygen elements in Figure 7f. 

**Figure 8:**
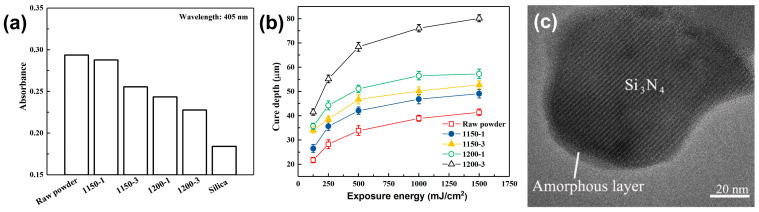
(**a**) The absorbance of Si_3_N_4_ samples and silica. (**b**) The cure depth of raw and oxidized Si_3_N_4_ samples. (**c**) TEM micrograph for a Si_3_N_4_ particle oxidized at 1150 °C for 1 h. Adapted from ref. [101].

In addition, Huang et al. [101] successfully synthesized Si_3_N_4_@SiO_2_ core–shell-structured particles by oxidizing Si_3_N_4_ particles at different temperatures and durations (1150 °C for 1 h and 1200 °C for 3 h). With increased oxidation, the particle absorbance decreased from approximately 0.3 to around 0.23, while the maximum curing thickness rose from less than 40 μm to about 80 μm, as shown in Figure 8a,b. Notably, Figure 8c illustrates that the SiO_2_ shell layer had a thickness in the nanometer range. It is clear that the sub-micron/nano-scale thickness of the oxide shell contributes to the improvement of the curing behavior of non-oxide ceramic VP slurry while significantly reducing the amount of impurities introduced into the slurry.

**Figure 9:**
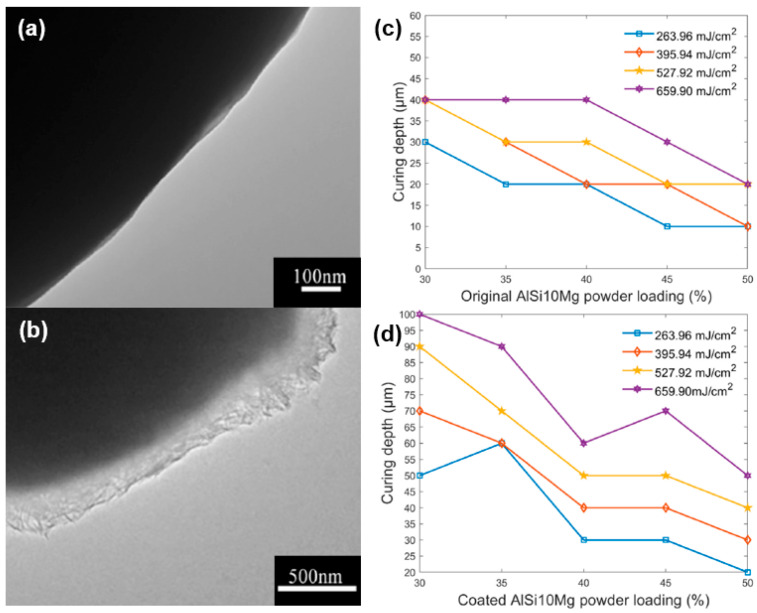
TEM micrograph for (**a**) the original AlSi10Mg particle and (**b**) the polystyrene–AlSi10Mg composite particle. Curing depth of slurries with different particles, (**c**) original AlSi10Mg particles and (**d**) polystyrene–AlSi10Mg composite particles. Adapted from ref. [102].

Furthermore, even though the raw material consists of metal particles rather than non-oxide ceramic particles, the work of Zhang et al. [102] also offers valuable insights. They synthesized AlSi10Mg@polystyrene particles by polymerizing styrene on the surface of AlSi10Mg particles using the BPO initiator at a heating temperature of 75 °C. Figure 9a,b illustrate the TEM micrograph for an original AlSi10Mg particle and a polystyrene-AlSi10Mg composite particle. It can be seen that the thickness of the polystyrene coating is only a few hundred nanometers. Comparatively, the maximum curing thickness of the slurry formulated from the uncoated particles was approximately 40 μm, whereas the slurry formulated from the coated particles exhibited a curing thickness of around 90 μm, as shown in Figure 9c,d.

Distinct from the use of inorganic materials as coatings, the polymers encapsulating the particle surface can be removed during debonding and sintering processes. As a result, the influence of low-extinction-capacity fillers terminates at the end of the molding process. This leads to a reduction in the quantity of impurity elements left in the final product, yielding a nearly pure phase product.

### 4.5. Incident Light Intensity and Critical Light Intensity

The logarithmic value is positive when the true number ($E_{0}/E_{crit}$) exceeds 1. This indicates that the incident light intensity $E_{0}$ surpasses the critical light intensity $E_{crit}$, enabling the curing process to commence. For a certain VP slurry, the $E_{crit}$ value is determined. In non-oxide ceramic VP slurries, the $E_{crit}$ value is notably higher than that of oxide ceramic slurries due to their higher light-extinction capacity. Consequently, a higher $E_{0}$ is required to initiate system curing.

**Figure 10:**
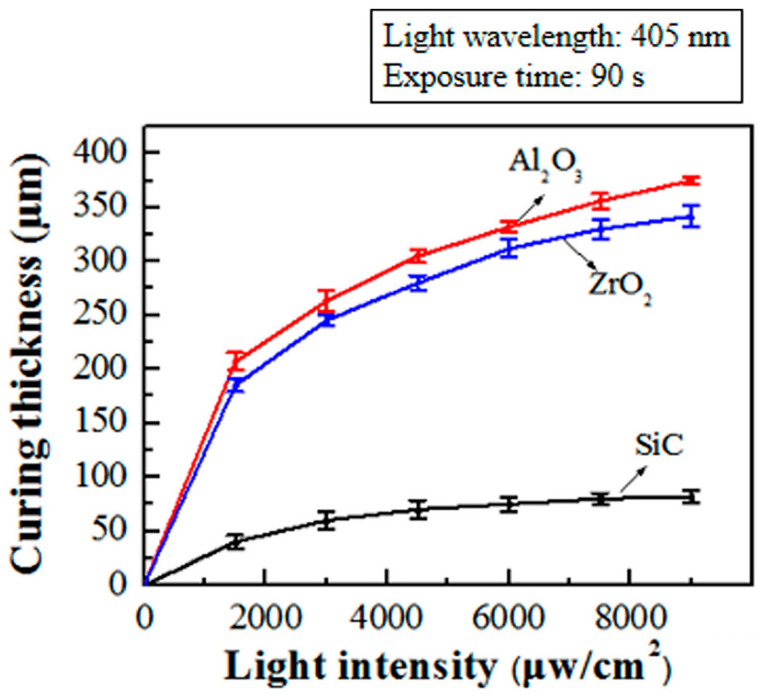
The relationship between light intensity and curing thickness. Adapted from ref. [49].

Ding et al. [49] configured VP slurries with a solid loading of 30 vol% using Al_2_O_3_, ZrO_2_, and SiC and investigated the relationship between light intensity and curing thickness. As shown in Figure 10, the curing thickness of the SiC slurry was significantly lower than that of the two oxide slurries at the same light intensity. As $E_{0}$ increased, the curing thickness inevitably experienced some growth. However, once $E_{0}$ reached a certain threshold, the growth rate diminished significantly and eventually stabilized. Further increasing $E_{0}$ at this point did not contribute significantly to an improved curing thickness. Moreover, excessively high incident light energy can cause resin carbonization, resulting in failure of the VP process. Additionally, portions of the slurry that have already cured impede light propagation, hindering further curing [49]. Therefore, while initially effective, relying on an increased light intensity to enhance curing thickness quickly loses its effectiveness as light intensity is further increased.

### 4.6. Reactivity of Photosensitive Resins

**Figure 11:**
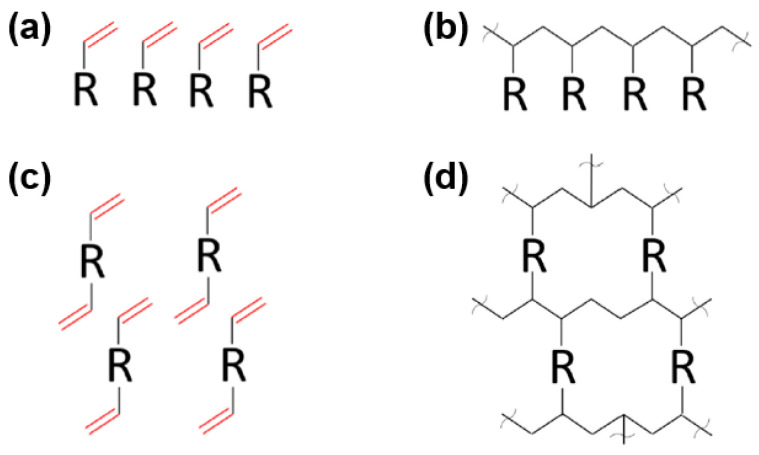
Comparison of mono and multifunctional monomers: (**a**) monofunctional monomers; (**b**) linear polymerization of monofunctional monomers; (**c**) multifunctional monomers; (**d**) network polymerization of multifunctional monomers. Adapted from ref. [103].

The main constituents of photosensitive resins typically include monomers or oligomers with varying functionalities, sometimes accompanied by non-reactive diluents. Figure 11 illustrates the impact of polymerization and crosslinking in both mono and multifunctional monomers. It is evident that resins with a greater reactivity exhibit a higher functionality, making it easier to swiftly form a highly crosslinked mesh structure [103].

However, resins with a high functionality also tend to have a higher viscosity, which hinders the preparation of VP slurries with a higher solid loading [85]. Additionally, high polymerization shrinkage results in elevated internal stresses during the curing process, potentially causing delamination and brittleness in the green parts [104,105]. Consequently, the introduction of diluents becomes necessary to regulate the viscosity and polymer shrinkage of the slurry. Table 3 provides examples of photosensitive resins and diluents with varying numbers of functional groups.

**Figure 12:**
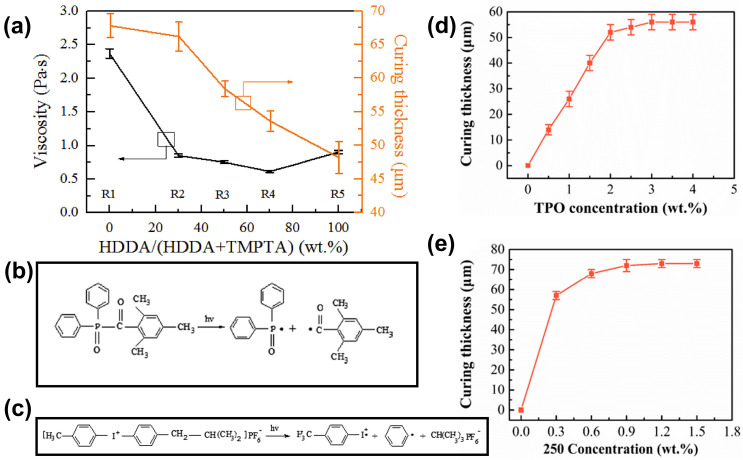
(**a**) Viscosity and curing thickness of SiC slurries with different monomers. Adapted from ref. [106]. (**b**) The initiate mechanism for free radical photoinitiator TPO, (**c**) the initiate mechanism for cationic photoinitiator 250, (**d**) the effects of TPO concentration on the curing thickness of the SiC slurry, and (**e**) the effects of 250 concentration on the curing thickness of the SiC slurry. Adapted from ref. [49].

Ding et al. [106] obtained SiC VP slurries by using two typical acrylate monomers compounded as bifunctional HDDA and trifunctional TMPTA. The mass ratios of HDDA/(HDDA+TMPTA) were varied as 0, 30, 50, 70, and 100 wt.%, resulting in slurries R1, R2, R3, R4, and R5, respectively. By increasing the mass ratio of TMPTA, the functionality of the resin system was increased. Under the same conditions used for all the other parameters, the curing thickness of the slurry increased as the incorporation of TMPTA increased. For instance, as shown in Figure 12a, the curing thickness rose from 48.2 μm for pure HDDA to 67.8 μm for pure TMPTA. This work demonstrates that increasing the reactivity of the photosensitive resin can effectively optimize the light-curing behavior of the slurries. The greater functionality of the resin system allows for more reactive sites, leading to improved polymerization and curing efficiency. As a result, the curing thickness of the slurry increases.

In addition to investigating the impact of resin functionality, Ding et al. [49] also examined the influence of photoinitiator type and dosage on the light-curing behavior of SiC slurries. Two commonly used initiators were selected: TPO, a free radical initiator; and 250, a cationic initiator. Figure 12b,c illustrate the initiate mechanisms for TPO and 250. The slurry containing the cationic initiator 250 exhibited a higher curing thickness compared to the one with TPO. This can be attributed to the fact that the cationic initiator is capable of generating both super-strong protonic acid and active free radicals. These species weaken the oxygen inhibition effect, thereby allowing for a more efficient curing process. In contrast, the free radical initiator may be more susceptible to the inhibitory effects of oxygen, leading to a lower curing thickness. Furthermore, the amount of initiator used in the slurry formulation was found to have an impact on the curing thickness. Figure 12d,e illustrate the effect of initiator (TPO/250) concentration on the curing thickness of a SiC slurry. Initially, as the initiator dosage increased, the curing thickness of the slurry showed a tendency to increase. This can be attributed to the increased availability of reactive species, which promotes a more complete polymerization reaction. However, there may be a saturation point beyond which further increases in initiator dosage do not significantly contribute to the curing thickness. This is because the photoinitiators can only receive a limited amount of light energy, which restricts their overall efficiency.

Obviously, the optimization of the light-curing behavior in this way is limited. As mentioned earlier, there is direct competition between the absorption of light by the ceramic particles and the liquid phase (i.e., photosensitive resin and photoinitiator). Consequently, the amount of light energy that the liquid phase can absorb without diminishing the extinction ability of the ceramic particles is restricted. Thus, the modulation of photosensitive resins and photoinitiators can only achieve a modest degree of optimization in light-curing behavior. Furthermore, increasing the functionality of the photosensitive resin and the activity of the photoinitiator introduces new challenges, such as an elevated viscosity of the slurry system, excessively rapid polymerization speed, and excessive internal polymerization stress.

## 5. Challenges and Prospects

As mentioned previously, to improve the curing behavior of non-oxide ceramic slurries and achieve a higher cure thickness, researchers have pursued various methods. While the existing improved methods have been able to increase cure thicknesses to some extent, there remains a need for further optimization and enhancement of the VP process for non-oxide ceramics. Based on the above discussion of the current development status, the following challenges and prospects can be summarized.

### 5.1. Expanding the Material System of Non-Oxide Ceramic VP Processes

Currently, research on non-oxide ceramics for VP processes is primarily focused on SiC and Si_3_N_4_. However, there exist numerous non-oxide ceramic materials with excellent properties that have not yet to be utilized in the VP process. For instance, boron carbide (B_4_C) is an ideal neutron absorber that is capable of absorbing a significant number of neutrons without producing radioactive isotopes, making it suitable for application in the nuclear industry [107,108,109]. Meanwhile, zirconium boride (ZrB_2_) exhibits exceptional and stable high-temperature performance, rendering it a promising candidate for aerospace high temperature-resistant materials [110,111,112]. Despite these advantages, the VP process of these materials remains underexplored. Therefore, it is imperative to expand the material system of non-oxide ceramic VP processes to fabricate complex-shaped parts with a high dimensional accuracy, ultimately enabling their application in various industries.

### 5.2. Coordinating Multiple Curing Mechanisms to Improve Curing Behavior

**Figure 13:**
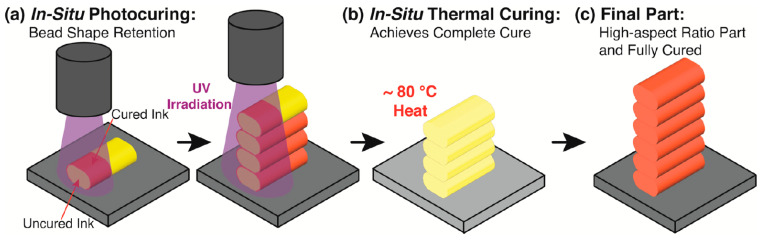
Schematic of the UV-ME printing of the dual-cure ink. (**a**) The initial light-curing process. (**b**) The subsequent heat-curing process. (**c**) The final part is fully cured and achieves the final mechanical properties. Adapted from ref. [114].

Conventional VP processes for non-oxide ceramics initiate cross-linking curing of the system solely through UV light-excited photoinitiators. Various methods for improving the curing behavior have been previously described. However, these methods have never been able to eliminate the limitation of light penetration depth, which in turn prevents higher curing thicknesses from being achieved. Therefore, some researchers have attempted to obtain more thorough curing by combining multiple curing mechanisms, such as coordinate thermal curing mechanisms with light-curing mechanisms [113,114,115,116]. Although the process used was material extrusion (ME) rather than vat photopolymerization, the work of Rau et al. [114] still provides a valuable reference. They introduced both photoinitiators (BAPO) and thermal initiators (AIBN) into commercially available resin (Ebecryl 230) and blended aluminum particles with a solid loading of 60 vol.% as feedstock for a UV-assisted ME process. The initial light-curing process can rapidly form a thin cured layer on the surface of the extruded material. The subsequent heat curing process allows for the complete curing of the slurry where UV light cannot reach. Figure 13 illustrates the process diagram of this work.

In Rau’s study, the heat is introduced by an overhead heater [114]. In the VP process, heat can be generated from two sources: either the heat released during the polymerization of photosensitive resins or the heat of the laser itself. However, it is important to note that although heat transfer is not limited by the depth of light penetration, it tends to be slower and requires longer processing times. In addition, heat transfer lacks a clear direction, which may affect print accuracy. Despite these shortcomings, it is evident that the coordination of multiple curing mechanisms can effectively circumvent the limitation of light penetration depth and thus improve the curing behavior of the VP process. Unfortunately, no studies have been conducted on the coordinate use of multiple curing mechanisms for non-oxide ceramic VP processes. Therefore, it is also an important challenge and opportunity for the future.

### 5.3. Explore Simulation Techniques of the Curing Process

To obtain a non-oxide ceramic slurry suitable for VP processes, an extensive exploration process is often necessary to optimize and adjust the terms influencing the light-curing behavior. Simultaneously, alterations in the ceramic particles result in changes to the process parameters, requiring readjustment of the optimal parameters. Westbeek [117,118] modeled the effects of the shape and distribution state of ceramic particles in the slurry on light transmission based on Maxwell’s electromagnetic equations. Although the considered model problems are two-dimensional, these studies lay the foundation for a modeling framework for three-dimensional problems. Obviously, conducting research on simulation techniques for the light-curing additive manufacturing process of non-oxide ceramics can significantly streamline the cumbersome process exploration, which has a positive effect on revealing the molding mechanisms and then feeding back to the additive manufacturing process.

## 6. Summary

In summary, improving the curing behavior of slurries is critical for non-oxide ceramic VP processes. While researchers’ investigations into various key factors can improve the curing behavior of slurries to some extent, several challenges and opportunities persist within this research domain. Further improvement of the curing behavior and gradual introduction of the prepared devices into practical applications would be a significant advancement in the research of non-oxide ceramic VP processes. For example, the curing behavior of non-oxide ceramics can be improved by coordinating multiple curing mechanisms to compensate for the lack of light penetration depth. Based on this, more kinds of non-oxide ceramics can be applied to the VP process and eventually introduced into practical applications. In addition, the study of simulation techniques for the light-curing process of non-oxide ceramics can greatly reduce the difficulty of exploring the VP process. The authors hope that this review could offer valuable insights and serve as a point of reference for further research on the light-curing behavior of non-oxide ceramics.

## Figures and Tables

**Table 1:** Band gap, bonding type, and color of silicon and its non-oxide and oxide.

Material	Band Gap (eV)	Color	Curing Thickness (μm)	Reference
SiC	3.2	Green	~50	[49]
Si_3_N_4_	5.3	Gray	~40	[50]
SiO_2_	8.9	White	~230	[48]

**Table 2:** Refractive index and curing thickness of typical ceramic particles used in the VP process.

Ceramic	Refractive Index	Curing Thickness (μm)	Solid Loading (vol%)	Reference
SiO_2_	1.56	~220	65	[48]
Al_2_O_3_	1.76	~150	50	[76]
ZrO_2_	2.05	~150	55	[77]
Si_3_N_4_	2.09	~40	30	[50]
SiC	2.55	~50	40	[49]

**Table 3:** Properties of resins and diluents used in the ceramic VP process.

Resins and Diluents	Functionality	Molar Mass (g/mol)	Viscosity (mPa·s)	Refractive Index	Reference
ACMO	1	141	12–15	1.512	[80,90]
IBOA	1	208	2–9	1.476	[91,92]
HDDA	2	226	5–10	1.455–1.457	[73,74,93]
PEGDA	2	308–508	15–65	1.463–1.467	[94,95]
TMPTA	3	296	80–140	1.474	[34,75]
DPHA	5/6	523–579	4000–7000	1.488–1.49	[96]
Glycerol	Non-reactive diluent	92	954	1.474	[97]
PEG	Non-reactive diluent	200–600	60–100	1.46–1.47	[98]
